# Altered Sarcomeric Structure and Function in Woody Breast Myopathy of Avian Pectoralis Major Muscle

**DOI:** 10.3389/fphys.2020.00287

**Published:** 2020-04-09

**Authors:** Jiao Liu, Eero Puolanne, Matthias Schwartzkopf, Anders Arner

**Affiliations:** ^1^College of Life Sciences, South-Central University for Nationalities, Wuhan, China; ^2^Thoracic Surgery, Department of Clinical Sciences, Lund, Faculty of Medicine, Lund University, Lund, Sweden; ^3^Department of Food and Nutrition, University of Helsinki, Helsinki, Finland; ^4^Deutsches Elektronen-Synchrotron, Hamburg, Germany

**Keywords:** avian muscle, hypertrophy, muscular dystrophy, muscle disease, sarcomere

## Abstract

The “Woody” or “Wooden” breast disease is a severe myopathy of pectoralis major muscle recently identified within rapidly growing broiler lines all around the world with a prevalence rate around 20%, or even higher. Although of significant ethical and economic impact, little is known regarding the structural and functional aspects of the contractile apparatus in the woody breast muscle. The aim of the present study was to determine physiological properties of the contractile system in the morphologically intact muscle fibers of focally damaged woody breast in comparison with normal muscle fibers to gain insight into the muscle function of the animal and possibly mechanisms involved in the disease development. Muscle samples were taken from woody breast (non-lesioned areas) and normal breast muscles from broilers. Length-tension curves, maximal active stress, maximal shortening velocity, calcium sensitivity, rate of tension development, lattice spacing and muscle biochemical composition were investigated on single skinned fibers. Sarcomeres of woody breast fibers were more compliant, which is very likely related to the wider spacing (18% wider compared to controls) between thick and thin filament. No differences were found in optimal sarcomere length (2.68 ± 0.04 *vs.* 2.65 ± 0.05 μm) nor in maximal active stress (116 ± 17 *vs.* 125 ± 19 mN mm^–2^). However, woody breast fibers had less steep descending arm as shown in length-tension curve. Woody breast muscle fibers had 40% bigger sarcomeric volume compared to controls. Content of contractile proteins (myosin and actin), and maximal shortening velocity were unchanged indicating that the growth in woody breast muscle fiber was associated with synthesis of new contractile units with unaltered kinetics. Calcium sensitivity was decreased in woody breast muscle fibers significantly. In conclusion, the results show that the rapid growth of muscle in woody breast disease is associated with significant structural and functional changes in the pectoralis major musculature, associated with alterations in the mechanical anchoring of contractile filaments.

## Introduction

Consumption of poultry meat has increased dramatically worldwide during recent years, due to its comparatively low environmental impact as well as a healthy nutritional profile, with low fat and high protein contents. In order to satisfy the high and increasing demands, breeders have been genetically selecting broilers toward a fast growth rate, and high breast muscle (*pectoralis major*) yield over the past few decades. As a consequence, the growth rate of broilers has increased during the last 50 years almost 3-fold, resulting in weights of more than 2 kg in less than 40 days, with breast weights of about 20% of the whole carcass weight (Petracci et al., 2015).

Unfortunately, the improvement of growth rate is accompanied by increasing prevalence rates of several muscular abnormalities in the breast muscle, such as pale, soft and exudative (PSE) like meat (Chen et al., 2018), white striping (Kuttappan et al., 2012) and the woody (also denoted wooden) breast condition (Sihvo et al., 2014). Compared to the other two abnormalities, the woody breast is a more recent problem and has already been reported by several big chicken meat producing countries worldwide. The prevalence rate is around 10–20% (Trocino et al., 2015) and has been shown even higher in some reports (Mutryn et al., 2015; Petracci et al., 2019). Woody breast muscles are easily identified by hand palpation having a hard consistency, with pale appearance, and in more severe cases, hemorrhages in lesion areas. Histological examination has revealed that the affected areas in woody breast muscle usually have moderate to severe myodegeneration and regeneration, such as muscle fiber necrosis, infiltration of inflammatory cells, fibrosis, fat accumulation and centralized nuclei (Sihvo et al., 2014). Besides the economic loss, woody breast myopathy gives rise to serious concerns regarding animal welfare (Norring et al., 2019). In view of the 10–20% or higher prevalence and the large number of slaughtered broilers worldwide (more than 45 billion per year worldwide, AVEC, 2011 report) woody breast would by far be the most common muscle disease in the world today.

Ever since woody breast was first reported as a broiler meat quality deterioration problem several years ago, studies have been done (see review by Petracci et al., 2019) in aspects including pathological description of the lesions (Sihvo et al., 2014), *post mortem* physical and chemical characteristics of affected breast muscle (Soglia et al., 2015), and the properties of the muscle during further food manufacturing (Mudalal et al., 2015). Based on the currently available literature, it is likely that the occurrence of the woody breast is related to the heavier breast muscles or hypoxia (Soglia et al., 2015; Baldi et al., 2017; Dalle Zotte et al., 2017; Papah et al., 2017; Petracci et al., 2019) suggesting that rapid growth is involved in the pathogenesis. On the muscle fiber level, woody breast affected muscle tends to have higher abnormal fiber prevalence, but to our knowledge, little is known regarding on the structural and functional aspects of the contractile apparatus within woody breast muscle fibers under physiological conditions. The proper assembly and organization of the basic contractile units in each myofibril have critical roles in the morphology and, most importantly, the function of muscle fibers. It is also important to note that the structure and mechanical function of the sarcomere can feedback on the growth and adaptation of the muscle structure/function, with significant contacts between the cytoskeleton and signaling pathways involved in the maintenance of the cellular structure (e.g., titin in the sarcomere, Lange et al., 2005; vimentin and desmin in the cytoskeleton, Paulin and Li, 2004; Soglia et al., 2020); dystrophin in the dystroglycan complex (Rahimov and Kunkel, 2013). It is therefore relevant to gain more knowledge regarding the structural characteristics and function of the contractile apparatus in the woody breast muscle, since it will help us to understand how this myopathy influences muscle function *in vivo*, and cast light on mechanisms underlying the muscle dystrophy disease development in general.

In the current study, we examined woody breast muscle from a muscle physiology perspective. Muscle samples were prepared from normal breast and non-lesioned areas of woody breast. Sarcolemma was permeabilized (skinned preparations) preserving the intracellular contractile machinery enabling analysis length-tension curves, maximal active stress and maximal shortening velocity. Sarcomeric structure was measured using small angle x-ray scattering. We report alterations in the mechanical properties, including altered sarcomere mechanics and structure, which would influence mechanical function *in vivo* and possibly be related to the induction of hypertrophic growth.

## Materials and Methods

### Sample Selection

Eight normal and eight woody breast broiler pectoralis major muscles were dissected from sixteen Ross 308 chickens at 32 days old of both genders from the regular slaughter line in a local commercial slaughterhouse (average animal slaughter weight ∼2.2 kg as given by the slaughterhouse). Both normal and woody breast muscles were obtained from the same cohort of slaughtered animals.

Woody breast affected carcasses were identified by palpation on the slaughter line at 15 min *post mortem* together with normal carcasses. After being excised, the status of the breast muscles was confirmed visually and by palpating according to the criteria described by Sihvo et al. (2016) based on the appearance of white and hard areas vs. normal color and texture. Since the current study focused on identifying early changes in the woody breast condition development, we included woody breast affected muscles with only focal lesions in the cranial area and less affected medial area. Comparisons were made with normal breast muscle obtained from non-affected animals. Samples for analysis were taken from the medial part of normal controls and woody breast affected muscles. The samples were immediately fixed for histology or treated with chemical skinning as described below.

The study was fully based on slaughterhouse material obtained from the normal food production slaughterhouse line, after the animals were slaughtered. The study does not involve any study of live animals. No interventions or examinations were done prior to the slaughter.

### Histological Analysis

Muscle bundles (10 mm long × 2 mm thick) were slightly stretched, pinned to cork plates and fixed in buffered 4% paraformaldehyde for 24 h at 4°C. After transfer to a 70% of ethanol solution, samples were trimmed to obtain both longitudinal and transverse profiles of the muscle fibers, embedded in paraffin, sectioned at 4 micrometers, stained with hematoxylin and eosin, for visualization of tissue and muscle structure, and examined by light microscopy.

### Skinned Fiber Preparations

Fiber bundles (10 mm long × 2 mm thick) were tied at both ends with silk thread, stretched to the approximate *in situ* length on plastic holders and chemically skinned (sarcolemma permeabilized) essentially as described by in Iorga et al. (2012). The skinned preparations were kept relaxed in ATP-containing solution with composition (in mmol L-1): K_2_EGTA 10, Na_2_ATP 7, MgAcetate 2, MOPS 10, and K-propionate 122, pH 7.0, with 50% glycerol at −20°C for further analysis.

The woody breast affected samples displayed a mixture of degenerating muscle and morphologically “normal” muscle areas. Since we aimed at examining early stages of the myopathy, we isolated single muscle fibers from the morphologically intact areas and mounted for subsequent analysis. Fibers with clear sarcomere patterns and homogeneous width along fiber axis were included in the experiments.

### Sarcomere Length Measurements

Each isolated single fiber was attached using cellulose acetate glue (Sigma-Aldrich) between a force transducer (AE801, Kronex) and a micromanipulator enabling length changes. Experiments were performed in standard relaxation solution which contained (in mmol L-1) K_2_EGTA 6, MgATP 5, free Mg^2+^ 0.5, phosphocreatine 15, MOPS buffer 20, DTE 2 and K-propionate to an ionic strength of 200, with creatine kinase 0.5–1 mg ml-1 at pH 7.0. Free [Ca^2+^] was approximately 1 nmol L-1 (pCa 9) (All chemicals were purchased in Sigma-Aldrich). For each animal, 4–5 fibers were analyzed.

### Length–Tension Relationship

To determine the relationship between fiber length and passive/active force, single fibers were mounted at slack length between a force transducer and a micro manipulator as described above, and repeatedly activated at room temperature using a solution with high Ca2+ (pCa (-log [Ca^2+^]) = 4.7), same composition as the relaxing solution, except that K_2_EGTA is replaced by K_2_CaEGTA) and relaxed in between at room temperature. Muscles were stretched between contraction steps, and passive and active forces were recorded at each length step. Fiber length (relative to slack) was determined using light microscopy. Optimal stretch for active force was 1.3 of the slack length, and fiber width was determined at that length and used to calculate the maximal active stress (i.e., force per cross sectional area). Two to three single fibers from each animal were measured. For each fiber the force response at each L/Ls step was first normalized by its maximal force. The data from the fibers of each animal was averaged, and used as representative in the statistical analysis.

### Fiber Width Determination

Fiber diameter at optimal stretch (1.3 relative to slack length, L/Ls) was determined from different single fibers (>50 fibers for both woody and normal beast group) during sarcomere length measurements, length-tension experiments, force velocity experiment and k_TR_ determinations using light microscopy and an ocular scale (Zeiss Axiovert 35, inverted microscope, 20 × objective with 2.5 × magnification and a 10 × ocular). Three measurements at different positions along the long axis were taken from each fiber and averaged. In total, 6–7 measurements of fiber diameter from each animal were obtained and the average of these measurements for each animal were calculated and compared.

### Determination of Force–Velocity Relationship, Stiffness and Rate of Tension Development

To obtain information on the kinetics of the actin-myosin interaction we determined the force-velocity relationship, i.e., the dependence of shortening velocity on force and the maximal shortening velocity (V_max_) at room temperature. Single fibers were mounted as described above between the force transducer and a motor, enabling rapid changes in muscle length and force via a computer-controlled feedback system (Aurora Scientific 802B, ON, Canada). A series of 14 force steps was applied at the plateau of a maximal contraction. Stable after-loaded force levels were obtained in about 15 ms. For each step, length and force signals were recorded during 300 ms. The afterload (Pa) and the isometric force (Po), as well as the mean shortening velocity (V, between 20 and 70 ms after the force step in muscle length ML s^–1^) were analyzed for each step. Force velocity curves were constructed from the relationship between V and relative force (Pa/Po). For each animal, 1 fiber was analyzed.

For each force step, the linear regression for the time period 20–70 ms (used to calculate velocity) was extrapolated to the time of release and the intercept of this line was used to determine the series elastic recoil.

In separate experiments the rate of tension development (k_TR_) was estimated using a protocol described by Brenner and Eisenberg (1986). The fibers were shortened by 28% and re-stretched to initial length after 30 ms. The apparent rate constant for the force development was estimated from half-time of the force increase.

### Calcium Sensitivity Measurement

In another series of measurements, two bundles around 0.2 mm in diameter from each animal sample were analyzed. The averaged result of the two bundles represented one animal. Preparations were attached to a force transducer (Kronex) and stretched to 1.3 times of slack length in relaxing solution. After an initial activation at pCa 4.7, a series of solutions with intermediate free [Ca^2+^], pCa: 9, 6.0, 5.7, 5.5, 5.2, and 4.7, calculated as described by Fabiato (1988), were used to activate the muscle. The maximal force obtained at pCa 4.7 was used to normalize each contraction in response at lower pCa values. A hyperbolic equation [F = c^h^/(EC${}_{50}^{\text{h}}$ + c^h^)] was fitted to the normalized force responses (F) at different free [Ca^2+^] (c), to determine the EC_50_ (concentration giving half maximal force) and the Hill coefficient (h, describing the steepness of the curve).

### SDS-PAGE Analysis

A muscle bundle (around 2 mg wet weight, including several muscle fibers) was dissected from the skinned preparations, rinsed and homogenized using glass homogenizers in 100 × volume of 1 × Laemmli sample buffer (Bio-Rad) containing 1% SDS and 50 mmol L^–1^ DTT and heated to 95°C for 10 min until all the tissue were completed dissolved. Samples were separated on 4–15% gradient gels (Mini-PROTEAN TGX Precast Gels, Bio-Rad). Three different volumes of the homogenates, including one woody and normal breast muscle sample, together with three different known amounts of albumin standard and a molecular weight standard were loaded on each gel. Gels were stained using PAGEBLUE Protein Staining Solution (Thermo Fisher) and the quantification of stained protein bands were done using a CHEMIDOC XRS and the QUANTITY ONE program (Bio-Rad, RRID: SCR_014280). For each lane, the intensity of the bands corresponding to myosin heavy chain (MHC, 220 kDa) and actin (42 kDa) were determined. For each homogenate, a linear regression curve was fitted to the relation between loaded volume, and the MHC and actin band intensity respectively. The ratio between this slope and the slope of the albumin standard was then used to calculate the protein content (μg mg^–1^ wet muscle weight) of the sample.

### Small Angle X-Ray Scattering

Small angle x-ray scattering (SAXS) was used to determine the lattice spacing of the normal and woody breast muscle samples. Skinned muscle preparations were mounted in relaxation solution in a cuvette with Kapton window and stretched to different relative length (sarcomere length). The SAXS experiments were performed at the beamline P03, at Petra III, DESY, Hamburg (Buffet et al., 2012). A beam with a wavelength of 0.95 Å and a size of 16 × 22 μm^2^ was used. The equatorial diffraction patterns were recorded with 0.1 to 1 s exposures using a PILATUS 300K detector (DECTRIS Ltd.) in 5.1 m distance as described previously (Liu et al., 2018). The signal was calibrated using rat tail collagen. A polynomial function was fitted to the equatorial intensity signal for background subtraction and the center of mass for the reflections was determined. The spacing of the equatorial 1.0 and 1.1 reflections were measured. Based on collagen calibration, d_10_ and d_11_ from the muscle samples were calculated which are related to the spacing between two adjacent thick filaments mathematically. Lattice volume (Vol) was determined as described by Millman (1998) by multiplying lattice area with sarcomere length (SL):

(1) $$\text{Vol}=2/\sqrt{3}\times \text{d}\,10^{2}\times \text{SL}$$

Vol was estimated from the slope of the relationship between sarcomere length and 1/(d10)^2^. The 1.1/1.0 intensity ratio was determined as an indicator of mass transfer from thick to thin filament, e.g., when cross-bridge attachment is initiated, an increase of 1.1/1.0 will be observed according to Millman (1998).

### Statistical Analysis

All data are given as mean ± SEM with number of animals indicated. Several muscle fibers were analyzed for each animal, the average was taken as a representative value for that animal. Statistical analyses were made using SigmaPlot 14 (RRID: SCR_003210) for Windows (Systat Software Inc.). Two-way ANOVA, and the Holm–Sidak method were used for comparisons of normal and woody breast fibers at stretches, L/Ls ≥ 1.8. Student’s *t*-test was applied after confirmation of normality (Shapiro–Wilk) and equal variance (Brown–Forsythe), and Mann–Whitney Rank Sum Test used for active tension comparison at L/Ls 1.6.

## Results

**FIGURE 1:**
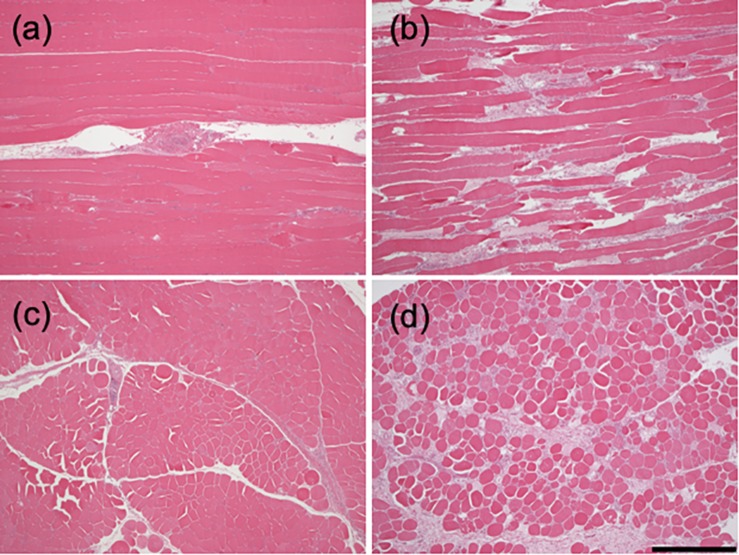
Histology of woody and normal breast muscle. Longitudinal **(a,b)** and cross-sections **(c,d)** of clinically normal pectoralis major muscle **(a,c)** woody breast **(b,d)** muscles (bar = 0.5 mm).

Figure 1 shows longitudinal- and cross-sections of samples from normal and woody breast muscles. The control muscle displays mainly well-organized muscle fibers with equal staining, with small areas of inflammatory infiltrates centered on larger blood vessels. In woody breast samples, discontinuities were found among the fibers in longitudinal sections accompanied by extensive fibrosis and inflammation. Transverse sectioning of muscle fibers clearly shows varying fiber diameters, presence of degrading fibers and a significant fibrosis. These morphological data thus show a clear dystrophy in the woody breast group and essentially normal structure in the control group. In the mechanical analysis, we examined morphologically intact fibers from woody breast affected muscle; possibly displaying early changes associated with disease development, and did not include severely damaged fibers. Therefore, only skinned fibers with clearly visible sarcomere patterns were included.

**FIGURE 2:**
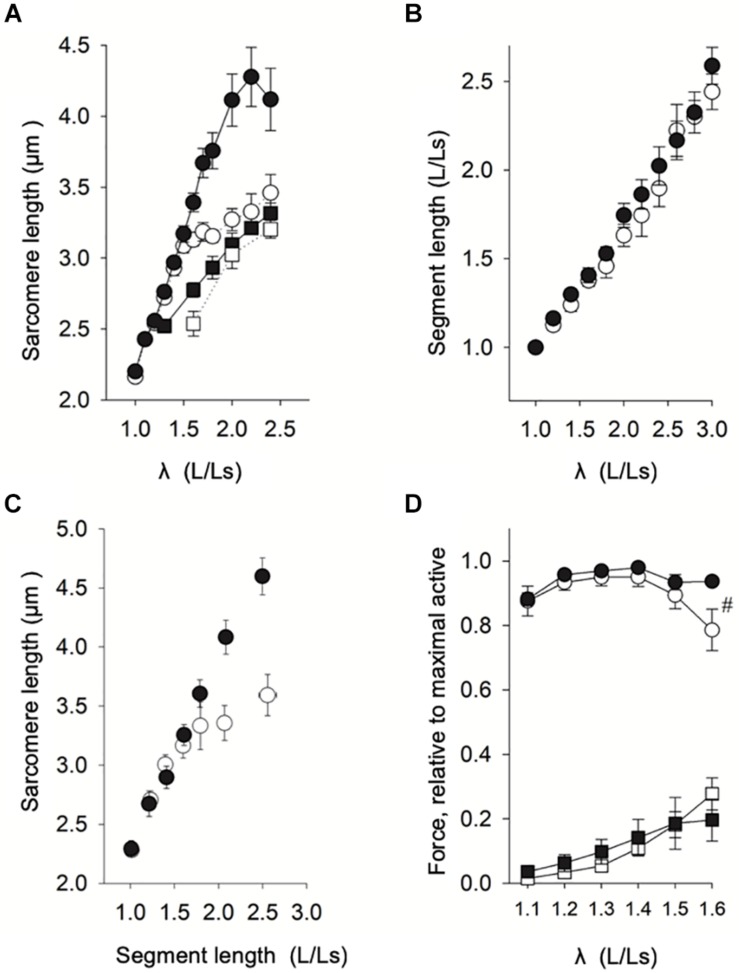
Length-force analysis of woody and normal breast muscle. **(A)** Relation between muscle length (related to slack length, Ls) and sarcomere length in normal breast (open circles) and woody breast (filled circles) muscles (data from eight animals, 4–5 fibers each, grouped according to L/Ls, each L/Ls: *n* = 6–30). Filled squares show the relation of woody breast fibers compressed by 20% (to the same fiber diameter as that of normal breast fibers) using 4% dextran T-500, and open squares show the relation of normal breast fibers compressed by 20% using 4% dextran T-500. For L/Ls values ≥ 1.8, the sarcomere length of the woody breast group was significantly longer compared to normal muscle fibers (*P* < 0.05, Two-way ANOVA). **(B)** Relation between segment length, i.e., the distance between two carbon dots adhered to the fiber surface (relative to slack length, Ls, ∼60 μm), and muscle fiber length (relative to slack fiber length). **(C)** Relation between sarcomere length and segment length (relative to Ls) in normal (open circles) and woody (filled circles) muscle fibers (data from six animals, 2–3 fibers each, grouped according to segment length/Ls, *n* = 6–13). **(D)** Active (circles) and passive (squares) length-force relationships of normal (open symbols) and woody breast (filled symbols) fibers (average data from eight animals, 2–3 fibers each, grouped according to L/Ls, each L/Ls: *n* = 6–8). # Indicates significant difference in active force at L/Ls = 1.6 (*P* < 0.05).

The first step was to investigate the relationship between sarcomere length and stretch of the fiber (given as muscle length/slack length, λ, L/Ls) in relaxed fibers from both groups. Figure 2A (including data from 6-30 fibers from 8 samples, grouped according to L/Ls) showed that for L/Ls values ≤ 1.7, no difference in sarcomere lengths between normal breast (open circles) and woody breast groups (filled circles) was observed. At L/Ls values ≥ 1.8, sarcomeres were longer in the woody breast groups (*P* < 0.05, two-way ANOVA) compared to the normal breast sarcomere lengths. In the control normal breast muscle, sarcomere length increased with stretch up to ∼1.5 L/Ls, reaching about 3.2 μm, and then increased less steeply with fiber length increase to about 3.4 μm at 2.5 L/Ls. In contrast, the sarcomere length of woody breast fibers increased with stretch and reached ∼4.2 μm at 2.4–2.5 L/Ls. These data suggest that passive structures limiting sarcomere extension during muscle fiber stretch are lacking or weaker in the woody breast group. We observed that upon extreme extension of fiber length, 70% of normal breast single fibers broke when L/Ls was above 2.6, whereas most of the woody breast fibers were able to be extended to 2.8-3.0 L/Ls. We therefore performed the stretch experiments up to 2.5 L/Ls. Since the stretch experiments of Figure 2A were done with length normalized to whole fiber length, we also examined if inhomogeneity along the fiber length or end-compliance, e.g., in the mounting, could affect the relationship between stretch and sarcomere length. Figure 2B shows that the extension of segments (about 60 μm, corresponding to about 25 sarcomeres) followed the extension of the whole fiber and Figure 2C shows that the difference in sarcomere extension between normal and woody breast fibers was observed also using this method.

As shown in Table 1, we found that woody breast fibers were about 20% wider in diameter compared to normal breast fibers. To study if the wider fiber diameter was related to the increased sarcomere extensibility when fibers were stretched (Figure 2A), we used osmotic compression (4% dextran added) to compress the woody breast fiber diameter to the same size as that of the control group (filled squares, Figure 2A, diameter 71 ± 5 μm, *n* = 6). Under these conditions, the sarcomere length response to stretch in the woody breast fibers became similar to that of the normal control fibers, suggesting that the altered coupling between fiber length and sarcomere length was related to the increase in fiber diameter. For comparison, the compression of normal breast fibers by 20% (diameter 63 ± 4 μm, *n* = 4) had minor effects on the relationship between stretch and sarcomere length (open squares, Figure 2A).

**TABLE 1:** Mechanical data and contractile protein expression.

	Normal breast	Woody breast	*P*
Optimal L/Ls	1.34 ± 0.05(8)	1.34 ± 0.03(8)	n.s.
Optimal sarcomere length, μm	2.68 ± 0.04(5)	2.65 ± 0.05(6)	n.s.
Fiber diameter, μm	73.0 ± 1.3(8)	88.0 ± 2.5(8)	***
Maximal active tension, mN	0.50 ± 0.08(8)	0.73 ± 0.08(8)	n.s.
Maximal active stress, mN mm^–2^	116 ± 17(8)	125 ± 19(8)	n.s.
Vmax, ML s^–1^	1.51 ± 0.08(4)	1.68 ± 0.16(5)	n.s.
Stiffness, ML (Pa/Po)^–1^	0.036 ± 0.003(6)	0.043 ± 0.004(6)	n.s.
k_TR_, s^–1^	19 ± 2(6)	10 ± 1(5)	**
EC_50_, pCa units	5.81 ± 0.02(6)	5.71 ± 0.03(6)	*
MHC, μg mg^–1^ wet weight	11.0 ± 1.4(5)	12.6 ± 1.4(6)	n.s.
Actin, μg mg^–1^ wet weight	9.0 ± 1.3(5)	8.8 ± 1.1(6)	n.s.
MHC/actin, molar ratio	0.27 ± 0.01(5)	0.31 ± 0.01(6)	*

Fiber diameter, sarcomere length and active force were determined at optimal stretch (relative to slack length, L/Ls) for active force. The maximal shortening velocity (Vmax) is given in muscle length/s (ML s^–1^). The series elastic stiffness is given as in muscle length/relative force [ML (Pa/Po)-1] where Pa and Po are afterload and isometric force, respectively. The rate of tension development (k_*TR*_) is given in s^–1^. The Ca^*2*+^ concentration where half of maximal force was developed (EC_50_) is given in pCa units. Number of animals is given in parenthesis. Myosin heavy chain (MHC) and actin were determined using quantitative SDS-PAGE. For fiber diameter: 6–7 fibers were analyzed per animal; for optimal lengths, maximal tension and stress: 2–3 fibers per animal; for Vmax and stiffness 1 fiber per animal; for MHC and actin: a sample containing several fibers per animal, loaded in different concentrations on the same gel; for k_*TR*_, 2 fibers per animal; for EC_50_, 2 fiber bundles per animal. *P < 0.05, **P < 0.01, ***P < 0.001, n.s., non-significant (Student’s *t*-test).

Our next step was to examine the length-force relationships, i.e., the passive and active force at different stretch (sarcomere length). As seen in Figure 2D, the relationship between stretch and active force was bell shaped with an optimal stretch of ∼1.3 L/Ls in both groups. The mechanical data are summarized in Table 1. The woody breast fibers had unchanged optimal stretch and optimal sarcomere length, but significantly increased fiber diameter at optimal stretch, compared to the normal controls. Both the maximal active force and calculated maximal active stress tended to be higher in the woody breast group, although not significantly different from the controls. We observed that at 1.6 L/Ls, the woody breast fibers had significantly (*P* < 0.05) higher active force compared to normal breast fibers in the descending limb of the active length-force curve (rightmost circles in Figure 2D). Passive force, in the relaxed muscle, reflecting properties of elastic components in parallel with contractile components, increased during extension of the fibers in a similar manner in the two groups (squares in Figure 2D).

**FIGURE 3:**
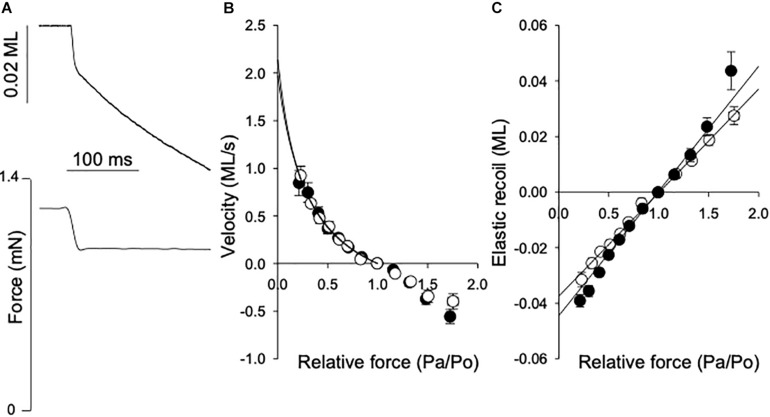
Force-velocity relationship and series elasticity. **(A)** Shows original traces of the fiber length shortening response (lower) to a rapid change in contraction force (top), from a normal breast muscle. The force step and length response are indicated in the figure. Shortening velocity = reduction in muscle length (ML)/shortening time (s). The afterload force (Pa) and reduction in muscle length were determined between 20 and 70 ms after the force step. **(B)** Shows the velocity (ML s-1) at different after loads related to the isometric force (Po). Data from 6 experiments were grouped according to Pa/Po and the data for shortening (i.e., velocity ≥ 0) were fitted by the hyperbolic Hill equation (Eq. 1). The equation was extrapolated to Pa/Po = 0 to determine the maximal shortening velocity, Vmax (fit to data in figure: normal breast, open symbols: 2.1; woody breast, filled symbols: 2.0 ML s^–1^). **(C)** Depicts the initial elastic recoil for different force steps. The data between 0.5 and 1.5 Pa/Po were fitted by a straight line [Normal breast, open symbols: 0.037; woody breast, filled symbols: 0.045 ML (Pa/Po)-1].

The maximal shortening velocity (V_max_) reflects the turnover of myosin-actin cross-bridges under unloaded conditions, mainly determined by the cross-bridge dissociation rate (Huxley, 1957). To determine this parameter, we performed isotonic quick release experiments, where the muscle was maximally activated to a stable isometric force (Po) and then exposed to a series of after loads (Pa), as illustrated in Figure 3A. The velocity (V) determined during the time period 20–70 ms after each release is plotted against relative after load (Pa/Po). In Figure 3B, data from 4-5 experiments were grouped according to after load and fitted with the Hill force-velocity equation in the following form:

(2) $$V=\frac{b\times \left(1-\frac{P\,a}{P\,o}\right)}{a+\frac{P\,a}{P\,o}}$$

V_max_ was calculated from the fitted parameters a and b as b/a. The mean values of V_max_ determined in individual muscle fibers are shown in Table 1. The mean values for parameter a were 0.38 ± 0.06 and 0.32 ± 0.08 and b 0.58 ± 0.11 and 0.52 ± 0.08, ML s^–1^, in normal and woody breast respectively, *n* = 4 and 5. No difference was found between normal and woody breast muscle fibers suggesting that although we have observed changes in woody breast muscle fibers, e.g., compliance, fiber width and lattice spacing (Figure 2 and Table 1), these differences are not associated with altered kinetics in the actin-myosin interaction when muscle is doing concentric work (i.e., shortening under load). It should be noted that the woody breast fibers appeared to be somewhat more compliant in the negative velocity region (Pa/Po > 1) of the force-velocity relationship, which might suggest altered behavior when muscle is doing eccentric work (i.e., stretched while contracting).

Results of stiffness and k_TR_ determination showed that woody breast fibers have more complaint sarcomeres compared to the controls. The isotonic quick release experiments enabled determination of the properties of the series elastic components using the initial length change after the force step. It should be noted that our setup enabled force steps complete within about 15 ms, but would reflect the series elastic components in the muscles. In Figure 3C the elastic recoil (SE) is plotted against afterload (Pa/Po). The data between 0.5 and 1.5 Pa/Po were fitted by a straight line, where the slope would be the inverse of the spring constant of a linear spring. The values in Table 1 show a slightly more compliant SE in the woody breast group compared to that of the normal breast muscle, although the mean values were not significantly different. In a separate series of experiments the rate of tension development (k_TR_) was estimated using a shortening, re-stretch protocol. A significantly lower rate was observed in the woody breast group (Table 1), since the series-elastic components are stretched during the force redevelopment, the data are consistent with a higher compliance associated with minor changes in cross-bridge kinetics in the woody breast group.

**FIGURE 4:**
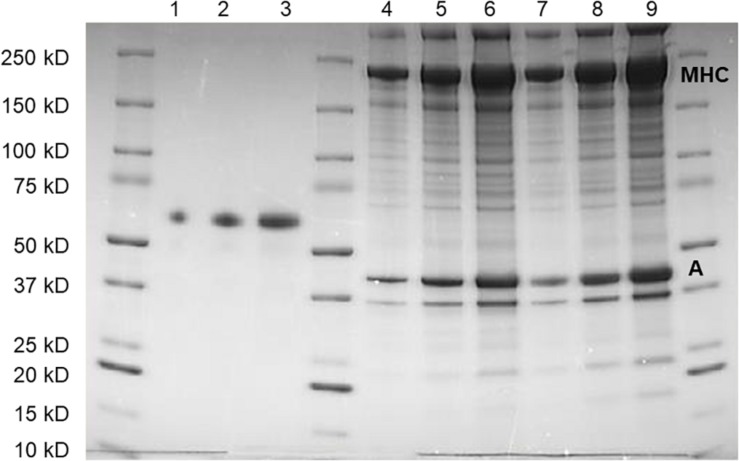
SDS-poly acrylamide gel separation of protein extracts from normal (Lanes 4–6) and woody breast (Lanes 7–9) together with albumin standard (Lanes 1–3). Three different volumes of samples and amount of albumin were loaded on each gel. Myosin heavy chain (MHC) and actin (A) were identified on the basis on their apparent molecular weights.

Contractile protein content was determined using quantitative SDS-PAGE (Figure 4) with Coomassie staining and different amounts loaded on each gel. We did not perform a detailed analysis of the protein composition, but no obvious differences in band appearance were observed on the gels between woody and normal breast samples, as shown in Figure 4. Using an albumin standard, we determined myosin heavy chain (Mw. ∼220 kD) and actin (Mw. ∼42 kD) contents, and related the contents to skinned fiber wet weight. No significant differences in myosin or actin were found between normal and woody breast samples (Table 1), although myosin content was slightly higher and the ratio of myosin to actin was significantly higher in the woody breast group.

**FIGURE 5:**
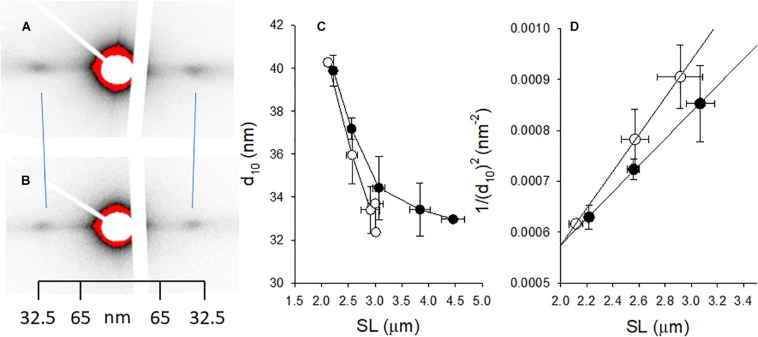
Small angle x-ray scattering of muscle fibers from normal control [**(A)** open symbols in **(C,D)** and woody breast] [**(B)** filled symbols in **(C,D)**]. **(A,B)** Show original recordings of x-ray patterns where the 1.0 equatorial reflections are indicated. Note inward movement of reflections in the woody breast group, showing wider lattice spacing. Calibration from collagen pattern is indicated. **(C)** Depicts the spacing of the 10 reflection (d_10_) and in **(D)** the 1/(d_10_)^2^ is plotted against the sarcomere length (SL), with straight lines fitted to the data (the slope is inversely related to lattice volume, ref: Millman, 1998). The estimated lattice volumes were 3.18 × 10^–3^ and 4.42 × 10^–3^ μm^3^ in normal and woody breast groups, respectively. *n* = 3–4.

Since fiber width was significantly increased in the woody breast group, we explored the possibility that also the volume of the contractile unit (sarcomere volume) was changed. We applied small angle x-ray scattering (SAXS) using synchrotron light. In principle, the regular lateral arrangement between contractile filaments results in interference patterns reflecting the filament distances. A wider spacing result in a smaller angle of the scattering. The equatorial reflections, the outer 1.1 and the inner 1.0, were recorded from both groups (Figures 5A,B) with a wider spacing in the woody breast group (i.e., inward movement of the reflections). Panel C shows a decrease in the lattice spacing with stretch of sarcomeres in the normal breast group, reflecting constant volume behavior. With increased sarcomere length, lattice spacing also declined in the woody breast samples but was generally wider. As seen also in Figures 2A,C, the woody breast samples could be extended to higher sarcomere lengths. Assuming a constant volume behavior the sarcomere volume could be calculated from data in the sarcomere range 2.2–3.0 μm, as shown in Figure 5D. The estimated sarcomere volume was about 40% bigger in the woody breast group compared to controls. The 1.1/1.0 intensity ratio, reflecting mass transfer between filaments, determined in the relaxed fibers at optimal stretch was low and not different between the two groups (normal breast 0.12 ± 0.02; woody breast 0.13 ± 0.03, *n* = 4 and 3). These data show that the muscles were fully relaxed and that major differences in the cross-bridge positioning in relaxed fibers can be excluded.

The force determination at different [Ca^2+^] determined at optimal stretch showed a significantly lower Ca^2+^ sensitivity in the woody breast group. Curve fit to the hyperbolic equation revealed no difference in the h parameters (normal breast: 3.1 ± 0.3; woody breast: 3.5 ± 0.2, *n* = 6 and 6) but significantly higher EC_50_ value (Table 1) reflecting the decreased Ca^2+^ sensitivity.

## Discussion

Woody breast muscle exhibits a pronounced myopathy with a loss of muscle fibers, which would significantly affect the contractile function of the breast muscle in the affected animals. The morphological changes have been reported previously (Sihvo et al., 2014, 2016, 2018; Velleman and Clark, 2015) and also our histological analysis shows that the affected animals were identified to have a clear muscle disease. To obtain information on early stages in the disease development prior to severe cellular degeneration, the present study focused on the structurally intact fibers from the woody breast muscle and reported alterations in sarcomeric structure and compliance.

The histological analysis of woody breast muscle revealed signs of degeneration and regeneration of muscle cells, including fiber splitting and fragmentation, increased number of rounded cellular cross-sections accompanied by fibrosis, which are consistent with previous observations (Sihvo et al., 2014, 2016, 2018; Velleman and Clark, 2015; Papah et al., 2017). Velleman et al. (2017) showed altered sarcomere organization in woody breast and Papah et al. (2017) observed irregular, disconnected and altered Z-disks by electron microscopy in woody breast affected muscle. Most likely these structural changes reflect an end stage of the myopathy which would be a consequence of earlier events in the disease process. In view of the tight links between the contractile system, the cytoskeleton and the membrane attachment sites, which are all involved in the maintenance of cellular function, it is possible that early changes in these structures can be involved in the induction of hypertrophy. The changes in sarcomeric structure, observed in the present study, could affect these cellular mechanical contacts. It might cause altered responses to external mechanical perturbations and body movement, and eventually initiate the dystrophy process via membrane injury and/or activation of cellular signaling for growth and degeneration.

In order to understand the development of the woody breast condition at a cellular level, it is worthwhile to analyze morphologically intact fibers rather than degenerated ones. Therefore, we set up criteria for fibers selected for analysis from woody breast samples, including only fibers with homogenous width along the fiber axis and clear sarcomere patterns. These fibers had significantly increased diameter at defined sarcomere lengths that are optimal for active force, which suggests that cellular hypertrophy is an important component in the woody breast condition, which is in agreement with previous reports on samples fixed for histology (Clark and Velleman, 2016; Dalle Zotte et al., 2017). It should be noted that our analysis is based on single fibers, whereas the behavior of the whole muscle will also be influenced by the loss of fibers and development of fibrosis in the later stage of the condition.

To know whether the increase in muscle cell size involved the addition of contractile components, we determined the contents of actin and myosin in woody breast muscle fibers. The amount of myosin and actin per milligram of muscle (i.e., concentration) were the same between woody breast and normal breast muscles. This shows that a net synthesis of sarcomeric proteins occurs during the hypertrophy in the woody breast, thereby maintaining the myosin and actin concentrations. To examine if the increase in contractile proteins was associated with an increase in parallel-coupled functional myofibrils, we determined active force development at optimal sarcomere length. The active stress (i.e., force/cross-sectional area) observed in our study of normal, control breast muscle (pectoralis major) is similar to values previous reported in the literature (Reiser et al., 1992, 1996). In woody breast fibers, the active stress was maintained, or even slightly increased, which shows that the increase in cell size is associated with a compensatory increase in the contractile components. It also shows that a loss of contractile function, as has been observed in some other forms of hypertrophic myopathy (e.g., myostatin deficiency, Amthor et al., 2007), is not a primary component in the degenerative process in woody breast disease.

Growth and re-arrangement of the sarcomeric units can affect the contractile performance in different ways. Our measurements of optimal sarcomere length and active stress suggest that the length of the sarcomere and the number of parallel-coupled actin-myosin cross-bridges per cross-section are maintained in the woody breast muscle fibers. Active force and contractile function can also be affected by the kinetics of the actin-myosin turn-over; a faster rate can lower active force, by decreasing the time a cross-bridge spends in force bearing states which would be associated with high ATPase and higher maximal shortening velocity (e.g., Rome et al., 1999). Myosin heavy chain (MHC) and myosin light chain (MLC) isoforms greatly influence the maximal shortening velocity in avian muscle (Lowey et al., 1993). The adult chicken pectoralis major muscle comprises mainly fast-twitch type IIB fibers with a fast myosin heavy chain type, with the addition of some slow and red-strip fibers located deeper in the tissue (Reiser et al., 1988, 1996; Remignon et al., 1995). We sampled from the breast muscle surface region in the 32-day-old broilers and report a maximal shortening velocity of about 1.6 muscle length/s (ML s^–1^) at 22°C. These V_max_ values for the normal chicken pectoralis major muscle are lower than the values for unloaded shortening of fast white muscle fibers previously reported by Reiser et al. (1996). The difference between the reports might reflect differences between techniques (isotonic quick release experiments vs. slack test) or possibly in the age or strain of chicken studied (ROS 308 vs. White Leghorn/New Hampshire). Importantly, no difference could be noted in our measurements between normal and woody breast muscles, suggesting that the contractile kinetics and the series coupling of sarcomeres are preserved. The rate of tension development was slower as predicted by the more compliant series elasticity located in the fiber or within the sarcomere, although the data do not exclude a slightly decreased rate of tension development. Modern selection for rapid growth of broiler is often accompanied by a shift to fast twitch fiber type that has bigger fiber width. However, our results of V_max_ and k_TR_ did not prove that woody breast muscle fibers are faster. Therefore, the observed increase in cross-sectional area in woody breast muscle cannot be explained by a shifting of fiber types to the faster ones.

Although the optimal sarcomere length and active contraction were similar in the normal and woody breast muscle, we observed significant changes in the structure and compliance of sarcomeres in the latter group. In the normal muscle, sarcomeres could not be extended in the relaxed state beyond about 3.2 μm when the fibers were stretched, suggesting the presence of structures limiting extreme sarcomere extension. In contrast, the woody breast fiber sarcomeres were significantly more extensible and could be extended almost linearly with stretch up to around 4.3 μm. This suggests that a factor limiting sarcomere extension is lacking or less prominent in the woody breast muscle fibers. The loss or conformational changes in cytoskeletal proteins acting as connecting components along sarcomere axis can be one possible reason for the compliance, but no obvious changes were seen in the present SDS-PAGE result. Previous protein analysis of woody breast muscle has not identified changes in myofibrillar proteins (Soglia et al., 2015). Therefore, to explain the altered sarcomere structure and compliance, a more detailed study on the changes within structural components and signaling factors is needed in the future.

The present study revealed enlarged sarcomeric volume in woody breast fibers compared to the controls by about 39%, which could influence the distance between adjacent thick and thin filaments at each defined sarcomere length. Muscle and sarcomeres act as constant volume systems, where elongation in sarcomere length results in a decreased cross-sectional area. Compression of lattice spacing reduces active force production but increases force during stretch of active muscles in frog fibers (Edman, 1999). In mammalian muscle, lattice compression has relatively fewer effects (Godt and Maughan, 1981; Zhao and Kawai, 1993; Liu et al., 2018), and only larger compression reduces active force (Maughan and Godt, 1981; Zhao and Kawai, 1993). It is thus unlikely that the difference in lattice spacing between normal and woody breast muscle fibers affected the active force generation at each stretch step. Nevertheless, the lattice spacing can influence the passive stress and extensibility of sarcomeres upon longer fiber length. Resistance might develop when adjacent filament surfaces touch each other during fiber elongation. As shown in Figure 5C, the minimum lattice spacing value (d_10_) is ∼ 33 nm in both woody and normal muscle fibers. This might reflect the minimal distance between filaments in the sarcomere where the myosin heads can be accommodated. The current study showed wider lattice spacing in woody breast sarcomeres, which could thus allow more extension before the minimal lattice spacing was reached. We compressed woody breast fibers laterally by osmotic force to the diameter of normal fibers and found that the length coupling between fiber and sarcomere was similar to that in the controls, reflecting a role of wider spacing. Since normal fibers were less affected by osmotic compression, the presented results demonstrated that the effect of lateral distance on sarcomeric compliance is operating in swollen sarcomeres, as in the woody breast muscle. We observed decreased calcium sensitivity in the woody breast group and a change in the lattice spacing might be a factor contributing to this phenomenon (Godt and Maughan, 1981). Avian muscles operate mainly in the sarcomere length range covering the ascending arm of the length-force relationship (Burkholder and Lieber, 2001), which did not differ between woody and normal breast muscle according to our measurements. However, a less steep descending arm was observed in the woody breast group where significantly higher force was observed at longer sarcomere length. This could be a consequence of the wider filament spacing in the woody breast group as discussed above (Williams et al., 2013).

Modern genetic selection for fast growth and high breast yield broilers is achieved by selecting animals with more profound muscle hypertrophy (Dransfield and Sosnicki, 1999), with average larger fiber cross-sectional areas (Berri et al., 2007) and/or increased fiber number (Scheuermann et al., 2004). In our study woody and normal breast samples were taken from a flock of the same hybrid and were thus genetically very homogeneous. This suggests that a genetic predisposition plays a role in the susceptibility to develop the syndrome and some environmental factors then trigger the onset of the syndrome in some animals. It has been shown that animals that grow even faster with more pronounced fiber enlargement are more prone to develop woody breast condition (Papah et al., 2017), which suggests that food intake or animal size are important triggering factors. It has been suggested that the woody breast condition is a consequence of hypoxia, increased oxidative stress and intracellular calcium alterations, which might be due to insufficient blood supply or impaired metabolism in the fast-growing animals (Mutryn et al., 2015; Petracci et al., 2019). It is difficult to ascertain whether these changes are primary or only consequence of the abnormal hypertrophic growth. The present study demonstrates the changes in the mechanical anchoring of contractile filaments in the woody breast muscles. This could affect the contractile properties and possibly activating signaling pathways associated with hypertrophic growth and ultimate muscle degeneration.

## Data Availability Statement

All data used in this study are available from the corresponding author upon reasonable request.

## Ethics Statement

The study was fully based on slaughterhouse material obtained from the normal food production slaughterhouse line, after the animals were slaughtered. The study does not involve any study of live animals. No interventions or examinations were done prior to the slaughter. The slaughterhouse providing the material (in Denmark) is operating according to the local legislation and animals are bread in normal food production.

## Author Contributions

JL and AA designed the study, performed most of the experiments, and conducted data analysis. JL, EP, and AA did sampling in slaughterhouse. JL, AA, and MS accomplished x-ray scattering experiment. JL and AA wrote the manuscript. All the authors participated during result discussion and manuscript revision before the submission.

## Conflict of Interest

The authors declare that the research was conducted in the absence of any commercial or financial relationships that could be construed as a potential conflict of interest.
